# 10-Methoxy­benzo[*g*]imidazo[1,2-*a*][1,8]naphthyridine-4-carbonitrile

**DOI:** 10.1107/S1600536809037544

**Published:** 2009-09-26

**Authors:** Andrii V. Tarasov, Tatyana A. Volovnenko, Noël Lugan, Yulian M. Volovenko

**Affiliations:** a Department of Chemistry, National Taras Shevchenko University, 64 Volodymyrska St, Kyiv 01601, Ukraine; b Laboratoire de Chimie de Coordination du CNRS, 205 route de Narbonne, 31077 Toulouse CEDEX 4, France

## Abstract

In the title compound, C_16_H_10_N_4_O, both the meth­oxy and nitrile substituents lie in the plane defined by the benzo[*g*]imidazo[1,2-*a*]-1,8-naphthyridine ring system, resulting in a nearly planar geometry for the entire mol­ecule (r.m.s. deviation of the non-H atoms from the mean plane is 0.044 Å). In the solid-state, the mol­ecules form a three-dimensional polymer through inter­molecular C—H⋯N and C—H⋯O hydrogen bonds. In addition, the packing mode results in stabilizing π–π stacking inter­actions between the asymmetric units.

## Related literature

For the synthesis of the title compound and a series of similar products, see: Volovnenko *et al.* (2009 ▶). For related compounds and their anti­bacterial or photophysical properties, see: Kondo *et al.* (1990 ▶); Gokhale & Seshadri (1987 ▶); Rajagopal & Seshadri (1991 ▶); Vijila *et al.* (2000 ▶). For the solid-state structures of other imidazonaphthyridine derivatives, see: Fun *et al.* (1996 ▶); Sivakumar *et al.* (1996*a* ▶,*b* ▶); Muthamizhchelvan *et al.* (2005*a* ▶,*b* ▶). For general metrical features within organic compounds, see: Allen *et al.* (1987 ▶). 
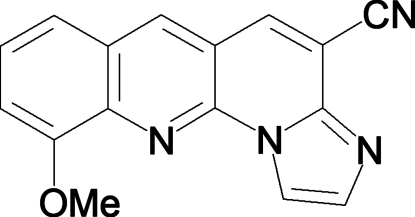


## Experimental

### 

#### Crystal data

C_16_H_10_N_4_O
*M* *_r_* = 274.28
Monoclinic, 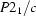

*a* = 7.710 (2) Å
*b* = 11.970 (2) Å
*c* = 13.340 (3) Å
β = 93.55 (3)°
*V* = 1228.8 (5) Å^3^
*Z* = 4
Mo *K*α radiation
μ = 0.10 mm^−1^
*T* = 180 K
0.40 × 0.40 × 0.35 mm

#### Data collection

Bruker APEXII diffractometer
Absorption correction: multi-scan (*SADABS*; Bruker, 2007 ▶) *T*_min_ = 0.95, *T*_max_ = 0.97
45253 measured reflections
3532 independent reflections
2507 reflections with *I* > 2σ(*I*)
*R*_int_ = 0.050

#### Refinement

*R*[*F* ^2^ > 2σ(*F* ^2^)] = 0.052
*wR*(*F* ^2^) = 0.164
*S* = 1.08
3532 reflections
191 parameters
H-atom parameters constrained
Δρ_max_ = 0.33 e Å^−3^
Δρ_min_ = −0.34 e Å^−3^

### 

Data collection: *APEX2* (Bruker, 2007 ▶); cell refinement: *SAINT* (Bruker (2007 ▶); data reduction: *SAINT*; program(s) used to solve structure: *SIR97* (Altomare *et al.*, 1999 ▶); program(s) used to refine structure: *SHELXL97* (Sheldrick, 2008 ▶); molecular graphics: *ORTEP-3 for Windows* (Farrugia, 1997 ▶) and *CAMERON* (Watkin *et al.*, 1993 ▶); software used to prepare material for publication: *WinGX* (Farrugia, 1999 ▶) and *publCIF* (Westrip, 2009 ▶).

## Supplementary Material

Crystal structure: contains datablocks I, global. DOI: 10.1107/S1600536809037544/fj2229sup1.cif

Structure factors: contains datablocks I. DOI: 10.1107/S1600536809037544/fj2229Isup2.hkl

Additional supplementary materials:  crystallographic information; 3D view; checkCIF report

## Figures and Tables

**Table 1:** Hydrogen-bond geometry (Å, °)

*D*—H⋯*A*	*D*—H	H⋯*A*	*D*⋯*A*	*D*—H⋯*A*
C8—H8⋯O1^i^	0.93	2.50	3.366 (3)	156
C3—H3⋯N1^ii^	0.93	2.62	3.394 (2)	141

Symmetry codes: (i) 
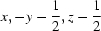
; (ii) 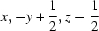.

**Table 2:** π–π stacking interactions (Å, °)

*Cg*_i_	*Cg*_j_	Centroid distance	Interplanar spacing ^i^	α^ii^	γ^iii^
*Cg*1	*Cg*2^iv^	3.487 (2)	3.322	2.68	15.42
*Cg*2	*Cg*1^iv^	3.487 (2)	3.361	2.68	17.68
*Cg*3	*Cg*3^iv^	3.710 (2)	3.382	0.00	24.27
*Cg*1	*Cg*4^v^	3.689 (2)	3.336	5.01	25.28
*Cg*4	*Cg*1^v^	3.689 (2)	3.397	5.01	22.98

Notes: (i) perpendicular distance between the centroid of the first ring and the plane of the second ring; (ii) dihedral angle between the plane of the first ring and the plane of the second ring; (iii) angle between the centroid of the first ring and the normal to the plane of the second ring; (iv) symmetry code: 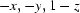; (v) symmetry code: 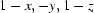. *Cg*1 is the centroid of atoms N1/C1/N2/C13/C14, *Cg*2 is the centroid of atoms N3/C12/C4–C6/C11, *Cg*3 is the centroid of atoms N2/C1–C4/C12 and *Cg*4 is the centroid of atoms C6–C11.

**Fig. 1.**
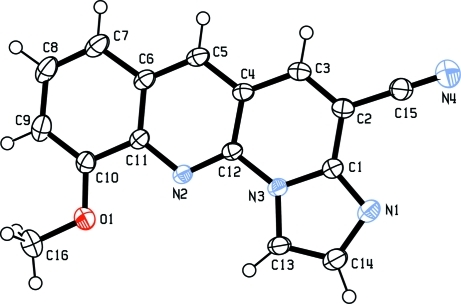
A perspective view of the title compound, with 50% probability displacement ellipsoids for non-H atoms.

**Fig. 2.**
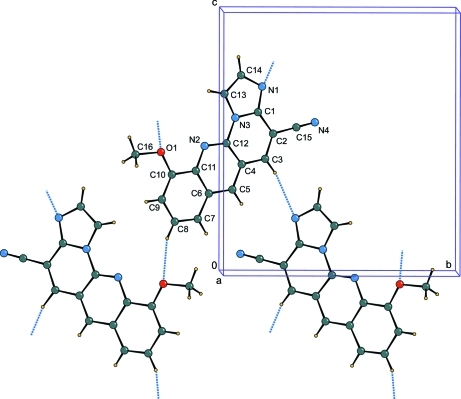
A packing diagram for the title compound, evidencing C—H···N et C—H···O hydrogen bonds (blue dotted lines).

**Fig. 3.**
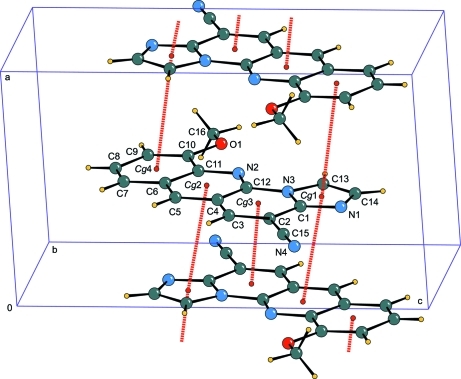
A packing diagram for the title compound, evidencing π-π stacking interactions (red dotted lines).

## supplementary crystallographic information

### Comment

New heterocyclic nitrogen-containing systems are always of great interest to synthetic as well as pharmaceutical organic chemists. We have recently reported an efficient and versatile route to benzo[*g*]imidazo[1,2-*a*]-1,8-naphthyridines upon thermal reaction of 2-chloroquinoline-3-carbaldehydes with 1-substitued hetarylacetonitriles (Volovnenko *et al.*, 2009). Here we report the crystal structure of the one of synthesized compound, namely, 10-methoxybenzo[*g*]imidazo[1,2-*a*]-1,8-naphthyridine-4-carbonitrile. It has been reported that products with similar structures possess not only antibacterial activity (Kondo *et al.*, 1990) but also interesting photophysical properties (Gokhale & Seshadri, 1987; Rajagopal & Seshadri, 1991; Vijila *et al.*, 2000).

Fig. 1 shows a perspective view of the asymetric unit of the title compound, including the labelling scheme. Selected bond distances and angles are given in Table 1. The benzo[*g*]imidazo[1,2-*a*]-1,8-naphthyridine core is almost planar (RMS deviation of C1>C14 and N1>N3 from mean plane is 0.035 Å). In addition, both methoxy and carbonitrile substituents attached to C(2) and C(11), respectively, are oriented in such a way that they both lay in heterocyle plane granting a nearly planar geometry to the entire molecule (RMS deviation of the all non-hydrogen atoms from mean plane is 0.044 Å). Bond distances and angles in the title compound are normal (Allen *et al.*, 1987) and compare well with other imidazonaphtyridines derivatives (Fun *et al.*, 1996; Sivakumar *et al.*.1996*a*,b; Muthamizhchelvan *et al.*, 2005*a*,b).

The crystal structure is stabilized by C—H···N and C—H···O intermolecular hydrogen bonds (Table 1) forming a three dimensional polymeric structure (Figure 2). In addition, the asymmetric units are seen to be stacked along the (100) axis with relatively short interplanar distances (Table 3) possibly allowing additional stabilization through π–π stacking interactions (Figure 3).

### Experimental

The title compound was synthesized by the reaction of 2-chloro-8-methoxyquinoline-3-carbaldehyde (2 mmol) with (1-benzyl-1*H*-imidazol-2-yl)acetonitrile (2 mmol) in dimethylformamide (3 ml). After refluxing for 1 h, the reaction mixture was left to stand overnight. The resulting crude solid was filtered, washed twice with acetone (10 ml) and dried. Yield: 96%. Crystals suitable for X-ray analysis were obtained by slow crystallization from hot dimethylformamide.

### Refinement

All H atoms attached to C atoms were fixed geometrically and treated as riding with C—H = 0.96 Å (methyl) or 0.93 Å (aromatic) with *U*_iso_(H) = 1.2*U*_eq_(C_arom_) or *U*_iso_(H) = 1.5*U*_eq_(C_methyl_).

### Crystal data

**Table:**

C_16_H_10_N_4_O	*F*(000) = 568
*M**_r_* = 274.28	*D*_x_ = 1.483 Mg m^−^^3^
Monoclinic, *P*2_1_/*c*	Mo *K*α radiation, λ = 0.71073 Å
Hall symbol: -P 2ybc	Cell parameters from 9999 reflections
*a* = 7.710 (2) Å	θ = 2.7–34.9°
*b* = 11.970 (2) Å	µ = 0.10 mm^−^^1^
*c* = 13.340 (3) Å	*T* = 180 K
β = 93.55 (3)°	Block, brown
*V* = 1228.8 (5) Å^3^	0.40 × 0.40 × 0.35 mm
*Z* = 4	

### Data collection

**Table:**

Bruker APEXII diffractometer	3532 independent reflections
Radiation source: sealed tube	2507 reflections with *I* > 2σ(*I*)
graphite	*R*_int_ = 0.050
φ scans	θ_max_ = 29.8°, θ_min_ = 2.3°
Absorption correction: multi-scan (*SADABS*; Bruker, 2007)	*h* = −10→10
*T*_min_ = 0.95, *T*_max_ = 0.97	*k* = −16→16
45253 measured reflections	*l* = −18→18

### Refinement

**Table:**

Refinement on *F*^2^	Primary atom site location: structure-invariant direct methods
Least-squares matrix: full	Secondary atom site location: difference Fourier map
*R*[*F*^2^ > 2σ(*F*^2^)] = 0.052	Hydrogen site location: inferred from neighbouring sites
*wR*(*F*^2^) = 0.164	H-atom parameters constrained
*S* = 1.08	*w* = 1/[σ^2^(*F*_o_^2^) + (0.0616*P*)^2^ + 1.0259*P*] where *P* = (*F*_o_^2^ + 2*F*_c_^2^)/3
3532 reflections	(Δ/σ)_max_ < 0.001
191 parameters	Δρ_max_ = 0.33 e Å^−^^3^
0 restraints	Δρ_min_ = −0.33 e Å^−^^3^
0 constraints	

### Special details

**Table:**

Geometry. All e.s.d.'s (except the e.s.d. in the dihedral angle between two l.s. planes) are estimated using the full covariance matrix. The cell e.s.d.'s are taken into account individually in the estimation of e.s.d.'s in distances, angles and torsion angles; correlations between e.s.d.'s in cell parameters are only used when they are defined by crystal symmetry. An approximate (isotropic) treatment of cell e.s.d.'s is used for estimating e.s.d.'s involving l.s. planes.
Refinement. Refinement of *F*^2^ against all reflections. The weighted *R*-factor *wR* and goodness of fit *S* are based on *F*^2^, conventional *R*-factors *R* are based on *F*, with *F* set to zero for negative *F*^2^. The threshold expression of *F*^2^ > σ(*F*^2^) is used only for calculating *R*-factors(gt) *etc*. and is not relevant to the choice of reflections for refinement. *R*-factors based on *F*^2^ are statistically about twice as large as those based on *F*, and *R*- factors based on all data will be even larger.

### Fractional atomic coordinates and isotropic or equivalent isotropic displacement parameters (Å^2^)

**Table:**

	*x*	*y*	*z*	*U*_iso_*/*U*_eq_	
C1	0.1160 (2)	0.16186 (14)	0.60289 (12)	0.0239 (3)	
C2	0.0449 (2)	0.22693 (14)	0.51977 (13)	0.0257 (3)	
C3	0.0661 (2)	0.19331 (15)	0.42439 (13)	0.0264 (4)	
H3	0.0201	0.2358	0.3708	0.032*	
C4	0.1593 (2)	0.09249 (14)	0.40567 (12)	0.0239 (3)	
C5	0.1800 (2)	0.05231 (15)	0.31021 (13)	0.0271 (4)	
H5	0.1352	0.0921	0.2546	0.033*	
C6	0.2686 (2)	−0.04838 (15)	0.29677 (12)	0.0262 (4)	
C7	0.2865 (3)	−0.09560 (17)	0.20044 (13)	0.0339 (4)	
H7	0.2407	−0.0588	0.1433	0.041*	
C8	0.3705 (3)	−0.19467 (18)	0.19165 (15)	0.0378 (5)	
H8	0.3800	−0.2260	0.1284	0.045*	
C9	0.4435 (3)	−0.25045 (16)	0.27749 (15)	0.0344 (4)	
H9	0.5029	−0.3173	0.2698	0.041*	
C10	0.4285 (2)	−0.20791 (14)	0.37173 (13)	0.0268 (4)	
C11	0.3374 (2)	−0.10535 (14)	0.38426 (12)	0.0232 (3)	
C12	0.2307 (2)	0.02653 (14)	0.48692 (12)	0.0222 (3)	
C13	0.2521 (2)	0.01703 (15)	0.67504 (13)	0.0282 (4)	
H13	0.3126	−0.0494	0.6869	0.034*	
C14	0.1924 (3)	0.08839 (16)	0.74444 (14)	0.0329 (4)	
H14	0.2065	0.0773	0.8135	0.039*	
C15	−0.0484 (2)	0.32689 (16)	0.54198 (14)	0.0307 (4)	
C16	0.5661 (3)	−0.36456 (17)	0.45063 (18)	0.0399 (5)	
H16A	0.4794	−0.4139	0.4207	0.060*	
H16B	0.6029	−0.3913	0.5164	0.060*	
H16C	0.6639	−0.3621	0.4095	0.060*	
N1	0.1082 (2)	0.17940 (14)	0.69986 (11)	0.0311 (3)	
N2	0.31704 (18)	−0.06777 (12)	0.47856 (10)	0.0231 (3)	
N3	0.20387 (18)	0.06431 (12)	0.58360 (10)	0.0233 (3)	
N4	−0.1221 (3)	0.40663 (16)	0.55947 (15)	0.0451 (5)	
O1	0.49489 (18)	−0.25543 (11)	0.45838 (10)	0.0338 (3)	

### Atomic displacement parameters (Å^2^)

**Table:**

	*U*^11^	*U*^22^	*U*^33^	*U*^12^	*U*^13^	*U*^23^
C1	0.0238 (8)	0.0251 (8)	0.0228 (8)	−0.0042 (6)	0.0013 (6)	0.0000 (6)
C2	0.0248 (8)	0.0261 (8)	0.0263 (8)	−0.0034 (6)	0.0010 (6)	0.0034 (6)
C3	0.0277 (8)	0.0265 (8)	0.0248 (8)	−0.0023 (6)	−0.0009 (6)	0.0060 (6)
C4	0.0253 (8)	0.0247 (8)	0.0215 (7)	−0.0043 (6)	0.0006 (6)	0.0037 (6)
C5	0.0313 (9)	0.0297 (8)	0.0201 (7)	−0.0062 (7)	−0.0001 (6)	0.0037 (6)
C6	0.0294 (8)	0.0281 (8)	0.0211 (8)	−0.0089 (7)	0.0025 (6)	−0.0002 (6)
C7	0.0440 (11)	0.0377 (10)	0.0205 (8)	−0.0124 (8)	0.0049 (7)	−0.0007 (7)
C8	0.0487 (12)	0.0401 (11)	0.0257 (9)	−0.0111 (9)	0.0107 (8)	−0.0086 (8)
C9	0.0401 (10)	0.0298 (9)	0.0344 (10)	−0.0070 (8)	0.0113 (8)	−0.0078 (7)
C10	0.0292 (8)	0.0236 (8)	0.0282 (8)	−0.0061 (6)	0.0051 (7)	−0.0006 (6)
C11	0.0249 (8)	0.0237 (8)	0.0213 (7)	−0.0074 (6)	0.0031 (6)	−0.0008 (6)
C12	0.0224 (7)	0.0242 (7)	0.0200 (7)	−0.0060 (6)	0.0011 (6)	0.0013 (6)
C13	0.0329 (9)	0.0296 (8)	0.0218 (8)	−0.0015 (7)	−0.0018 (6)	0.0046 (6)
C14	0.0385 (10)	0.0368 (10)	0.0234 (8)	−0.0033 (8)	0.0014 (7)	0.0012 (7)
C15	0.0314 (9)	0.0321 (9)	0.0285 (9)	−0.0003 (7)	0.0019 (7)	0.0026 (7)
C16	0.0434 (11)	0.0258 (9)	0.0509 (13)	0.0023 (8)	0.0056 (9)	−0.0012 (8)
N1	0.0360 (8)	0.0337 (8)	0.0239 (7)	−0.0032 (6)	0.0048 (6)	−0.0023 (6)
N2	0.0261 (7)	0.0234 (7)	0.0199 (6)	−0.0041 (5)	0.0016 (5)	0.0002 (5)
N3	0.0260 (7)	0.0237 (7)	0.0199 (6)	−0.0025 (5)	0.0000 (5)	0.0009 (5)
N4	0.0515 (11)	0.0399 (10)	0.0444 (10)	0.0086 (9)	0.0066 (8)	−0.0008 (8)
O1	0.0423 (8)	0.0264 (6)	0.0330 (7)	0.0034 (6)	0.0050 (6)	0.0004 (5)

### Geometric parameters (Å, °)

**Table:**

C1—N1	1.315 (2)	C9—C10	1.368 (3)
C1—N3	1.382 (2)	C9—H9	0.9300
C1—C2	1.436 (2)	C10—O1	1.360 (2)
C2—C3	1.354 (2)	C10—C11	1.429 (2)
C2—C15	1.436 (3)	C11—N2	1.354 (2)
C3—C4	1.434 (2)	C12—N2	1.319 (2)
C3—H3	0.9300	C12—N3	1.394 (2)
C4—C5	1.380 (2)	C13—C14	1.361 (3)
C4—C12	1.424 (2)	C13—N3	1.375 (2)
C5—C6	1.402 (3)	C13—H13	0.9300
C5—H5	0.9300	C14—N1	1.384 (3)
C6—C7	1.418 (2)	C14—H14	0.9300
C6—C11	1.425 (2)	C15—N4	1.142 (3)
C7—C8	1.360 (3)	C16—O1	1.423 (2)
C7—H7	0.9300	C16—H16A	0.9600
C8—C9	1.412 (3)	C16—H16B	0.9600
C8—H8	0.9300	C16—H16C	0.9600
			
N1—C1—N3	111.76 (15)	O1—C10—C11	114.95 (15)
N1—C1—C2	129.36 (16)	C9—C10—C11	119.84 (17)
N3—C1—C2	118.86 (15)	N2—C11—C6	122.89 (16)
C3—C2—C1	120.12 (16)	N2—C11—C10	118.69 (15)
C3—C2—C15	122.18 (16)	C6—C11—C10	118.41 (15)
C1—C2—C15	117.70 (16)	N2—C12—N3	117.42 (14)
C2—C3—C4	120.31 (16)	N2—C12—C4	125.71 (15)
C2—C3—H3	119.8	N3—C12—C4	116.86 (15)
C4—C3—H3	119.8	C14—C13—N3	105.12 (16)
C5—C4—C12	116.60 (16)	C14—C13—H13	127.4
C5—C4—C3	122.82 (15)	N3—C13—H13	127.4
C12—C4—C3	120.55 (15)	C13—C14—N1	111.80 (16)
C4—C5—C6	120.19 (16)	C13—C14—H14	124.1
C4—C5—H5	119.9	N1—C14—H14	124.1
C6—C5—H5	119.9	N4—C15—C2	179.7 (2)
C5—C6—C7	122.25 (17)	O1—C16—H16A	109.5
C5—C6—C11	117.77 (15)	O1—C16—H16B	109.5
C7—C6—C11	119.95 (17)	H16A—C16—H16B	109.5
C8—C7—C6	119.94 (18)	O1—C16—H16C	109.5
C8—C7—H7	120.0	H16A—C16—H16C	109.5
C6—C7—H7	120.0	H16B—C16—H16C	109.5
C7—C8—C9	120.70 (18)	C1—N1—C14	104.37 (15)
C7—C8—H8	119.6	C12—N2—C11	116.81 (14)
C9—C8—H8	119.6	C13—N3—C1	106.94 (14)
C10—C9—C8	121.13 (19)	C13—N3—C12	129.77 (15)
C10—C9—H9	119.4	C1—N3—C12	123.28 (14)
C8—C9—H9	119.4	C10—O1—C16	116.67 (15)
O1—C10—C9	125.21 (17)		

### Hydrogen-bond geometry (Å, °)

**Table:**

*D*—H···*A*	*D*—H	H···*A*	*D*···*A*	*D*—H···*A*
C8—H8···O1^i^	0.93	2.50	3.366 (3)	156
C3—H3···N1^ii^	0.93	2.62	3.394 (2)	141

Symmetry codes: (i) *x*, −*y*−1/2, *z*−1/2; (ii) *x*, −*y*+1/2, *z*−1/2.

### Table 2 π–π stacking interactions (Å, °)

**Table:**

*Cg*_i_	*Cg*_j_	Centroid distance	Interplanar spacing ^i^	α^ii^	γ^iii^
*Cg*1	*Cg*2^iv^	3.487 (2)	3.322	2.68	15.42
*Cg*2	*Cg*1^iv^	3.487 (2)	3.361	2.68	17.68
*Cg*3	*Cg*3^iv^	3.710 (2)	3.382	0.00	24.27
*Cg*1	*Cg*4^v^	3.689 (2)	3.336	5.01	25.28
*Cg*4	*Cg*1^v^	3.689 (2)	3.397	5.01	22.98

Notes: (i) perpendicular distance between the centroid of the first ring and the plane of the second ring; (ii) dihedral angle between the plane of the first ring and the plane of the second ring; (iii) angle between the centroid of  the first ring and the normal to  the plane of the second ring; (iv) symmetry code: -x, -y, 1-z; (v) symmetry code: 1 - x, -y,1 - z. *Cg*1 is the centroid of atoms N1/C1/N2/C13/C14, *Cg*2 is the centroid of atoms N3/C12/C4–C6/C11, *Cg*3 is the centroid of atoms N2/C1–C4/C12 and *Cg*4 is the centroid of atoms C6–C11.
